# Prevalence and correlates of physical disability and functional limitation among community dwelling older people in rural Malaysia, a middle income country

**DOI:** 10.1186/1471-2458-10-492

**Published:** 2010-08-18

**Authors:** Noran N Hairi, Awang Bulgiba, Robert G Cumming, Vasi Naganathan, Izzuna Mudla

**Affiliations:** 1 Department of Social and Preventive Medicine, Faculty of Medicine, University Malaya, Kuala Lumpar, Malaysia; 2 School of Public Health, University of Sydney, Sydney, Australia; 3 Julius Centre University of Malaya, Faculty of Medicine, University of Malaya, Kuala Lumpar, Malaysia; 4 Centre for Education and Research on Ageing, Concord Hospital, Australia; 5 ANZAC Research Institute, Concord Hospital, University of Sydney, NSW, Australia; 6 Ministry of Health, Malaysia

## Abstract

**Background:**

The prevalence and correlates of physical disability and functional limitation among older people have been studied in many developed countries but not in a middle income country such as Malaysia. The present study investigated the epidemiology of physical disability and functional limitation among older people in Malaysia and compares findings to other countries.

**Methods:**

A population-based cross sectional study was conducted in Alor Gajah, Malacca. Seven hundred and sixty five older people aged 60 years and above underwent tests of functional limitation (Tinetti Performance Oriented Mobility Assessment Tool). Data were also collected for self reported activities of daily living (ADL) using the Barthel Index (ten items). To compare prevalence with other studies, ADL disability was also defined using six basic ADL's (eating, bathing, dressing, transferring, toileting and walking) and five basic ADL's (eating, bathing, dressing, transferring and toileting).

**Results:**

Ten, six and five basic ADL disability was reported by 24.7% (95% CI 21.6-27.9), 14.4% (95% CI 11.9-17.2) and 10.6% (95% CI 8.5-13.1), respectively. Functional limitation was found in 19.5% (95% CI 16.8-22.5) of participants. Variables independently associated with 10 item ADL disability physical disability, were advanced age (≥ 75 years: prevalence ratio (PR) 7.9; 95% CI 4.8-12.9), presence of diabetes (PR 1.8; 95% CI 1.4-2.3), stroke (PR 1.5; 95% CI 1.1-2.2), depressive symptomology (PR 1.3; 95% CI 1.1-1.8) and visual impairment (blind: PR 2.0; 95% CI 1.1-3.6). Advancing age (≥ 75 years: PR 3.0; 95% CI 1.7-5.2) being female (PR 2.7; 95% CI 1.2-6.1), presence of arthritis (PR 1.6; 95% CI 1.2-2.1) and depressive symptomology (PR 2.0; 95% CI 1.5-2.7) were significantly associated with functional limitation.

**Conclusions:**

The prevalence of physical disability and functional limitation among older Malaysians appears to be much higher than in developed countries but is comparable to developing countries. Associations with socio-demographic and other health related variables were consistent with other studies.

## Background

The world's population is aging. The most rapid growth of older people is seen in the developing world [1]. At present, developed nations are experiencing relatively little change in the increase in older people as compared to the developing nations (middle income and low income countries) [1]. Malaysia is an example of such a population. The proportion of older Malaysians (age 60 years and above) will grow from 6.3% (1.4 million) in 2000 to 12% (4.9 million) by 2030, doubling in proportion but more than tripling in number [2].

Malaysia's economy has changed from agriculture to one that is supported by manufacturing, and high technology industry. As a result of this, the younger population are migrating to the urban areas, seeking employment. The older population, being less likely to migrate, are left behind to look after themselves. In 2000, 8% of Malaysian rural population comprised older people age 60 years and above as compared to only 5% in the urban population[2]. Malaysian health care delivery is provided by the government and private sectors. Health care in the rural areas is delivered through a network of health facilities supported by an organized system of referral. Mobile teams make regular visits to pockets of population in remote areas. Even though significant progress has been made in establishing an extensive health care delivery system for Malaysian rural population, the recent National Health and Morbidity Survey III in 2006 revealed that prevalence of hypertension, hypercholesterolemia, being underweight and smoking is much higher among the rural population than their urban counterparts[3]. A World Health Organization (WHO) study also revealed that Malaysian elderly residing in rural areas experienced greater financial hardship and express more need for health services than those in urban areas[4].

Physical disability and functional limitation are common among older people [5], leading to adverse consequences such as dependency and institutionalisation. Older people's ability to function independently is important, as physical disability and functional limitation have profound public health implications with increased utilization of health care and a need for supportive services and long term care [6]. This highlights the need to study the prevalence of physical disability and functional limitation especially in a rural underserved population of older adults.

Many studies on physical disability and functional limitation have been carried out in developed countries [7]. However, data are sparse for developing countries. We conducted a cross sectional study with the aim of determining the prevalence of physical disability and functional limitation in a developing middle income country and comparing these estimates to what has been reported in other countries. This study also aimed to determine the factors associated with physical disability and functional limitation in our Malaysian population.

## Methods

### Sample and Procedure

The participants were rural older people aged 60 years and above from the Alor Gajah Older People Health Survey (AGOPHS). This is a community survey of the physical and mental health of rural older people in Malaysia. In Malaysia 8% of the rural population are older people as compared to 5% of the urban population. AGOPHS was conducted between May 2007 and November 2008. This survey was conducted in Mukim Masjid Tanah, one of the sub-districts in Alor Gajah. Alor Gajah is a rural community situated in the northwest region of the State of Malacca. Eligible participants were those who had lived in Masjid Tanah for at least 12 months and who were 60 years of age and older; age was confirmed by their identity card. Exclusion criteria were non-Malaysian citizens, older people residing in nursing homes and admitted to hospitals. Survey participants were identified from a comprehensive community list. This list was developed by the Village Development Security Committee, a government institution at the village level. This organization administers and manages the villages and collects population and village profile annually. Interviews were conducted in participant's own home and participants were invited to the Health Clinic for the clinical assessments. Older people who missed the appointment were contacted twice in the subsequent weeks. The interviews and clinical assessments were conducted by five medical students. The medical students were given three day training by the study coordinator. The first day covered background to the AGOPHS and survey administration. The second and third days focused on the clinical assessments: the performance based functional limitation using the Tinetti Performance Oriented Mobility Assessment Tool and visual screening.

As majority of older people in the rural areas converse in Malay, the AGOPHS questionnaire was design in a bi-lingual (Malay and English) manner. The questionnaire had been pre-tested prior to the survey. The validated Malay version of Geriatric Depression Scale (GDS) and Elderly Cognitive Assessment Questionnaire (ECAQ) was used in this survey. Certain terminology and items in the questionnaire were made available in the languages of the main ethnic groups in Malaysia; Hokkien and Cantonese for the Chinese and Tamil for the Indians.

### Definition and measurement of physical disability and functional limitation

Two physical function measures were used in the analysis: performance-based functional limitation and self-reported physical disability. The Tinetti Performance Oriented Mobility Assessment Tool is a measure of functional limitation that assesses older people's gait and balance abilities [8,9]. Briefly, the participants began the assessments seated in a hard, straight-backed, armless chair. Participants' balance abilities were assessed by performing manoeuvres such as sitting in chair, rising from chair, immediate standing balance (first three to five seconds after standing), further standing balance, balance with eyes closed, turning balance, ability to withstand displacement when nudged on sternum, neck turning, one leg standing balance, back extension, reaching up, bending down and finally sitting down. For gait assessment, participants were asked to stand with the examiner in an obstacle-free hallway. Participants used their usual walking aid and were asked to walk down the hallway at their usual pace. Participants' gaits were observed for initiation of gait, step height, step length, step symmetry, step continuity, path deviation, trunk stability, walk stance and turning while walking. For manoeuvres such as path deviation, trunk stability, walk stance, the examiner walked behind the participants and for others, next to the participants. Participants were then asked to walk back at a "more rapid than usual but safe pace" using usual walking aids. Each activity was scored 0-1 or 0-2, where a score of 0 meant inability to perform the activity and a score of 1 or 2 meant ability to perform the activity. The maximum score for the gait component was 12 points and for the balance component it was 16 points. Participants with scores of less than 12 (for gait) or less than 16 (for balance) were defined as having performance based functional limitation.

Physical disability was determined by the ten-item Barthel Index. This is an assessment of patients' level of independence in activities of daily living (ADL) [10]. The ten ADL items assessed were feeding, bathing, dressing, grooming, toileting, bladder control, bowel control, transfer from bed to chair, walking and stair climbing. For this study, physical disability was defined as needing help in one or more of these ADL activities. For comparison with other studies, physical disability was also determined based on six Katz ADL items related to self-care (feeding, dressing, bathing, toileting, transferring and walking) [11-14] and five items related to self-care (feeding, dressing, bathing, toileting and transferring) [15-19].

### Socio-demographic and health related variables

Data on the following socio-demographic characteristics were collected by face-to-face interview: age, gender, ethnicity, education level, marital status, living arrangements and social support. Ethnic group was self reported and categorised as Malays, Chinese, Indians and Others. Information on the following health related variables was obtained: cognitive function, presence of chronic conditions, depressive symptomology, weight and height measurement and presenting visual acuity. Cognitive function was assessed using the Elderly Cognitive Assessment Questionnaire (ECAQ) [20]. ECAQ has been shown to be a valid tool for assessment of cognitive impairment among older people living in developing countries [20]. ECAQ scores range from 0-10. A score of 0 to 4 indicates probable cognitive impairment, 5 to 6 borderline and 7 and above as normal. Respondents were asked about the presence of chronic diseases using the following question: "Has your doctor ever told you that you suffer from.......(disease)?" The disease include: diabetes, epilepsy, hypertension, heart attack, coronary or myocardial infarction, angina, congestive heart failure, chronic lung disease, asthma, stroke and arthritis. Depressive symptomology was assessed using the short version of the Geriatric Depression Scale 15 item (GDS) [21]. Scores ranges from 0-15, with scores of six or more indicate depressive symptomology. Visual Acuity, height and weight were assessed at the Masjid Tanah Health Clinic and the Health Promotion Centre. Presenting visual acuity (PVA) was assessed using a standard metric Snellen Chart of E type or alphabets at 6 meters. Participants' presenting visual acuity (PVA) was ascertained with them wearing their habitual optical corrections (spectacles). The WHO definition of visual impairment defines mild or moderate visual impairment as PVA of less than 6/18 but equal to or better than 3/60 [22]. Blindness is defined as PVA of less than 3/60 in the better eye [22]. Height and weight were measured with the participants in light clothing and without shoes. Body mass index was calculated as weight (kg)/height (m) ^2^.

### Statistical Analysis

The SAS version 9.1 (SAS Institute, Inc., Cary, NC, USA) was used for all analyses. Participants with cognitive impairment identified through scores of less than five based on the Elderly Cognitive Assessment Questionnaire (ECAQ), were removed from the analysis (n = 27). The prevalence of six item ADL dependence and five item ADL dependence was estimated to allow comparison with prevalence of ADL disability reported in other studies. For comparison purposes, the prevalence of disability among older population in other studies was age standardized to our population using the direct standardization method. We also used direct standardization for comparing prevalence of physical disability and functional limitation across the three ethnic groups in our study Malays, Chinese and Indians, using the Malays as the standard population.

Due to the high prevalence of physical disability and functional limitation, prevalence ratios (PR) were calculated instead of odds ratio (OR) [23]. All analyses were carried out using SAS Proc Genmod's log binomial regression and Poisson regression with robust variance (when binomial regression models did not converge) [24]. Two sets of univariate analyses were performed using chi-square tests:-first, analyses were done to identify associations between the ten item physical disability and socio-demographic and health related variables and, second, similar analysis were done with functional limitation. Then, multivariate Poisson regression with robust variance was performed to test which of the socio-demographic and health-related variables were independently associated with physical disability and functional limitation. Variables with clinical and statistical significance (*p *value of < 0.25 in the univariate analysis) were included in the model.

### Ethics

Ethics approval was granted by the Medical Ethics Committee, University Malaya Medical Centre, Kuala Lumpur and the Ministry of Health, Malaysia. Informed (verbal) consent was obtained from all participants.

## Results

### Characteristics of study participants

Of the 907 eligible older people who were approached, 765 completed to the questionnaire and physical examination, giving a response rate of 84.3%. Non response was predominantly due to unavailability after two repeated invitations. The overall sample was representative of the older population in Alor Gajah Melaka [25]. Table 1 shows characteristics of the study sample. Participants ranged in age from 60 to 99 years (mean age of 69 ± 7 years). Thirty three of participants were men and 94% had some form of education. Women (16%) were more likely to live alone than men (4%). More women were cognitively impaired (5%) than men (1%). Over half of the participants had at least one self reported medical condition, with similar proportions among men and women.

**Table 1:** Characteristics of study population (n = 765) by sex

Variables	Men		Women		All	
	n	(%)	n	(%)	n	(%)
Age group						
60-64	111	(38.8)	174	(36.3)	285	(37.2)
65-69	73	(25.5)	99	(20.7)	172	(22.5)
70-74	54	(18.9)	105	(21.9)	159	(20.8)
≥ 75	48	(16.8)	101	(21.0)	149	(19.5)
						
Ethnic Group						
Malays	205	(71.7)	376	(78.5)	581	(76.0)
Chinese	43	(15.0)	66	(13.8)	109	(14.2)
Indians and Others	38	(13.3)	37	(7.7)	75	(9.8)
						
Education level						
No formal education	16	(5.6)	189	(39.3)	205	(26.8)
Primary education	220	(76.9)	256	(53.4)	476	(62.2)
Secondary education	43	(15.0)	28	(5.9)	71	(9.3)
Tertiary education	7	(2.5)	6	(1.2)	13	(1.7)
						
Marital status						
Married	252	(88.1)	232	(48.4)	484	(63.3)
Widowed	33	(11.5)	240	(50.1)	273	(35.7)
Others (single and divorced)	1	(0.4)	7	(1.5)	8	(1.0)
						
Living Arrangements						
Living alone	12	(4.2)	75	(15.7)	87	(11.4)
Living with others	274	(95.8)	404	(84.3)	678	(88.6)
						
Cognitive Assessment						
Normal	265	(92.7)	394	(82.3)	659	(86.1)
Borderline cognitive impaired	17	(5.9)	62	(12.9)	79	(10.4)
Probably cognitive impaired	4	(1.4)	23	(4.8)	27	(3.5)
						
Social support						
At risk of isolation	50	(17.5)	111	(23.2)	161	(21.0)
Not at risk of isolation	236	(82.5)	368	(76.8)	604	(79.0)
						
Presence of chronic disease						
None	100	(35.0)	149	(31.1)	249	(32.5)
One chronic disease	152	(53.1)	264	(55.1)	416	(54.4)
More than one chronic disease	34	(11.9)	66	(13.8)	100	(13.1)
						
Depressive Symptomology						
Yes	58	(20.3)	116	(24.2)	174	(22.7)
No	228	(79.7)	363	(75.8)	591	(77.3)
						
BMI (weight/height^2^)						
Normal (25.0-29.9)	104	(36.9)	164	(35.1)	268	(35.7)
Underweight (≤ 24.9)	138	(48.9)	195	(41.8)	333	(44.5)
Overweight (≥ 30.0)	40	(14.2)	108	(23.1)	148	(19.8)

### Prevalence of Physical disability and Functional limitation

Prevalence rates for physical disability and functional limitation are shown in Table 2. Overall, 24.7% reported needing help in at least one of the 10 ADLs in the Barthel Index. The prevalence of disability based on at least one item of the six item ADL scale was 14.4% and prevalence of disability based on at least one item in the five ADL scale was 10.6%. The prevalence of functional limitation was 19.5%

**Table 2:** Prevalence of Physical Disability and Functional Limitation among Older People in Malaysia (n = 738)

Variables	N	10 item ADL Dependence		6 item ADL dependence		5 item ADL dependence		Functional Limitation	
**Overall**									
≥ 60	738	24.7	(21.6, 27.9)	14.3	(11.9, 17.1)	10.6	(8.5, 13.1)	19.5	(16.8, 22.5)
≥ 65	458	37.3	(32.9, 41.8)	19.9	(16.5, 23.9)	15.6	(12.6, 19.2)	27.7	(23.8, 31.9)
≥ 70	288	44.8	(39.2, 50.6)	25.3	(20.6, 30.7)	19.8	(15.6, 24.8)	36.0	(30.7, 41.7)
≥ 75	139	49.6	(41.1, 58.2)	32.9	(25.5, 41.2)	24.5	(17.8, 32.6)	48.3	(40.1, 56.6)
									
**Age group**									
60-64	280	6.1	(3.6, 9.5)	4.9	(2.8, 8.0)	3.6	(1.8, 6.7)	5.6	(3.4, 9.1)
65-69	170	22.9	(17.0, 30.1)	10.5	(6.5, 16.3)	7.1	(3.9, 12.3)	12.8	(8.4, 18.9)
70-74	149	38.3	(30.5, 46.6)	18.4	(12.8, 25.5)	14.8	(9.7, 21.7)	24.5	(18.2, 32.1)
75 and over	139	49.6	(41.1, 58.2)	32.9	(25.5, 41.2)	24.5	(17.8, 32.6)	48.3	(40.1, 56.6)
									
**Male**									
≥60 (overall)	282	20.6	(16.1, 25.9)	12.2	(8.8, 16.7)	8.9	(5.9, 13.0)	14.7	(10.9, 19.5)
60-64	110	7.3	(3.4, 14.3)	5.4	(2.2, 11.9)	4.6	(1.7, 10.8)	4.5	(1.7, 10.7)
65-69	71	19.7	(11.6, 31.2)	13.7	(7.1, 24.2)	8.5	(3.5, 18.1)	13.7	(7.1, 24.2)
70-74	54	33.3	(21.5, 47.6)	16.7	(8.4, 29.8)	12.9	(5.8, 25.5)	18.5	(9.7, 31.9)
75 and over	47	38.3	(24.9, 53.6)	20.8	(11.0, 35.4)	14.9	(6.7, 28.9)	35.4	(22.6, 50.7)
									
**Female**									
≥60 (overall)	456	27.2	(23.2, 31.6)	15.6	(12.5, 19.2)	11.6	(8.9, 15.0)	22.3	(18.7, 26.4)
60-64	170	5.3	(2.6, 10.1)	4.7	(2.2, 9.4)	2.9	(1.1, 7.9)	6.3	(3.4, 11.3)
65-69	99	25.3	(17.3, 35.2)	8.1	(3.8, 15.8)	6.1	(2.5, 13.2)	12.1	(6.7, 20.6)
70-74	95	41.1	(31.2, 51.6)	19.2	(12.4, 28.4)	15.8	(9.4, 25.0)	27.6	(19.6, 37.4)
75 and over	92	55.4	(44.7, 65.7)	38.8	(29.3, 49.2)	29.4	(20.6, 39.3)	54.5	(44.3, 64.3)

The overall prevalence of disability (10 item ADL, 6 item ADL and 5 item ADL) and functional limitation increased with advancing age. The prevalence of needing help in at least one of the ten ADLs of the Barthel Index increased from 6% in those aged 60-64 years, to 50% of those aged 75 years and older. The prevalence of functional limitation rose for 6% in those aged 60 to 64 years to 48% in the 75 and above aged group.

Overall, the prevalence of both self-reported physical disability and objective measurement of functional limitation was higher in women than in men. (see Table 2).

**Figure 1:**
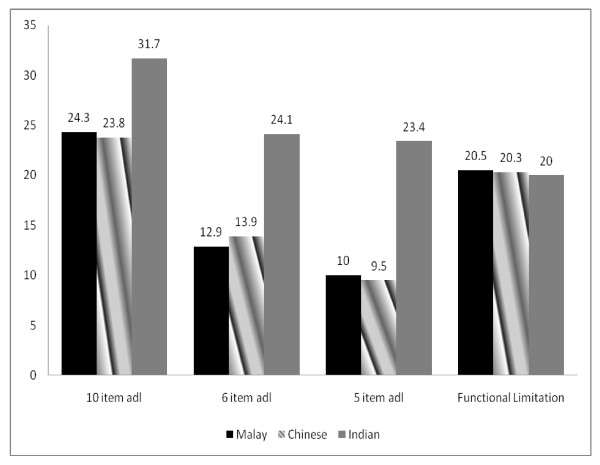
**Prevalence of poor physical function by ethnic group among older people in rural Malaysia**.

Among the three ethnic groups, Indians had the highest prevalence of physical disability (10 item ADL, 6 item ADL and 5 item ADL) compared to the Malays and Chinese (see Figure 1). However, the prevalence of functional limitation was similar across all ethnic groups. We analysed the correlations between performance based functional limitation and self reported physical disability among the different ethnic groups. The correlation coefficient for Malays, Chinese and Indians were 0.32, 0.29 and 0.14 respectively.

### Socio-demographic and Health Correlates

The univariate analyses (Table 3) showed that advancing age, being female, having low education (no formal education or only had primary education), self-report of one or more chronic medical conditions, having depressive symptomology and presence of visual impairment (mild to moderate and blindness) were associated with physical disability. In addition to these socio-demographic and health related variables, being at risk of isolation was associated with functional limitation (Table 4). Table 5 presents the multivariate associations between physical disability and functional limitation, and socio-demographic and health related variables. Significant independent associations for physical disability were found for advanced age, (≥ 75 years: prevalence ratio (PR) 7.9; 95% CI 4.8-12.9), presence of diabetes (PR 1.8; 95% CI 1.4-2.3), presence of stroke (PR 1.5; 95% CI 1.1-2.2), having depressive symptomology (PR 1.3; 95% CI 1.1-1.8) and visual impairment (blind: PR 2.0; 95% CI 1.1-3.6). Variables independently associated with functional limitation were: advanced age, (≥ 75 years: PR 3.0; 95% CI 1.7-5.2) being female (PR 2.7; 95% CI 1.2-6.1), presence of arthritis (PR 1.6; 95% CI 1.2-2.1) and having depressive symptomology (PR 2.0; 95% CI 1.5-2.7).

**Table 3:** Univariate analysis of variables associated with 10 items ADL dependence

Variables	Physical Disability present		Physical Disability absent		Unadjusted prevalence ratio	
	n	(%)	n	(%)	PR	(95% CI)
Age group						
60-64	17	(6.1)	263	(93.9)	1.0	(Reference)
65-69	39	(22.9)	131	(77.1)	3.3	(2.7, 4.0)
70-74	57	(38.3)	92	(61.7)	5.9	(4.4, 8.1)
≥75	69	(49.6)	70	(50.4)	10.8	(7.1, 16.3)
						
Sex						
Male	58	(20.6)	224	(79.4)	1.0	(Reference)
Female	124	(27.2)	332	(72.8)	1.8	(1.1, 3.0)
						
Ethnic Group						
Malays	135	(24.3)	420	(75.7)	1.00	
Chinese	25	(23.2)	83	(76.8)	1.3	(0.8, 2.1)
Indians and Others	22	(29.3)	53	(70.7)	1.4	(0.7, 3.0)
						
Education level						
Secondary and Tertiary education	21	(25.3)	62	(74.7)	1.0	(Reference)
Primary education	93	(19.8)	376	(80.2)	2.0	(1.3, 3.2)
No formal education	68	(36.6)	118	(63.4)	2.8	(1.4, 5.7)
						
Marital status						
Married	112	(23.8)	359	(76.2)	1.0	(Reference)
Widowed	69	(26.5)	191	(73.5)	1.2	(0.7, 1.9)
Others (single and divorced)	1	(14.3)	6	(85.7)	1.3	(0.6, 2.6)
						
Living Arrangements						
Living with others	158	(24.2)	495	(28.2)	1.0	(Reference)
Living alone	24	(28.2)	61	(71.8)	1.2	(0.8, 1.7)
						
Social support						
Not at risk of isolation	136	(23.2)	450	(76.8)	1.0	(Reference)
At risk of isolation	46	(30.3)	106	(69.7)	1.3	(1.0, 1.7)
						
Presence of chronic disease						
None	32	(13.1)	213	(86.9)	1.0	(Reference)
One chronic disease	101	(25.2)	300	(74.8)	4.1	(2.9, 6.0)
More than one chronic disease	49	(53.3)	43	(46.7)	8.4	(4.8, 14.5)
						
Diabetes						
No	95	(17.9)	437	(82.1)	1.0	(Reference)
Yes	87	(42.2)	119	(57.8)	2.4	(1.9, 3.0)
						
Arthritis						
No	125	(21.0)	469	(79.0)	1.0	(Reference)
Yes	57	(39.6)	87	(60.4)	1.9	(1.5, 2.4)
						
Stroke						
No	166	(23.6)	539	(76.4)	1.0	(Reference)
Yes	16	(48.5)	17	(51.5)	2.1	(1.4, 3.0)
						
Depressive Symptomology						
No	122	(21.1)	455	(78.9)	1.0	(Reference)
Yes	60	(37.3)	101	(62.7)	1.8	(1.4, 2.3)
						
Presenting visual acuity						
Normal	130	(22.5)	449	(77.5)	1.0	(Reference)
Mild to moderate visual impairment	42	(33.3)	84	(66.7)	1.7	(1.1, 2.5)
Blind	10	(30.3)	23	(69.7)	2.1	(1.2, 3.8)
						
BMI (weight/height2)						
Normal	65	(25.0)	195	(75.0)	1.0	(Reference)
Underweight	79	(24.8)	240	(75.2)	1.0	(0.7, 1.4)
Overweight	35	(24.3)	109	(75.7)	1.0	(0.6, 1.6)

**Table 4:** Univariate analysis of variables associated with functional limitation

Variables	Functional limitation present		Functional limitation absent		Unadjusted prevalence ratio	
	n	(%)	n	(%)	PR	(95% CI)
Age group						
60-64	15	(5.4)	265	(94.6)	1.0	(Reference)
65-69	21	(12.4)	149	(87.7)	4.1	(3.1, 5.4)
70-74	36	(24.2)	113	(75.8)	8.2	(5.4, 12.6)
≥ 75	65	(46.8)	74	(53.2)	16.7	(9.5, 29.2)
						
Sex						
Male	41	(14.5)	241	(85.5)	1.0	(Reference)
Female	96	(21.1)	360	(78.9)	2.1	(1.17, 4.1)
						
Ethnic Group						
Malays	107	(19.3)	448	(80.7)	1.0	(Reference)
Chinese	22	(20.4)	86	(79.6)	0.6	(0.4, 1.0)
Indians and Others	8	(10.7)	67	(89.3)	0.5	(0.2, 1.1)
						
Education level						
Secondary and Tertiary education	8	(9.6)	75	(90.4)	1.0	(Reference)
Primary education	78	(16.6)	391	(83.4)	2.8	(1.7, 4.6)
No formal education	51	(27.4)	135	(72.6)	4.6	(2.1, 9.9)
						
Marital status						
Married	80	(17.0)	391	(83.0)	1.0	(Reference)
Widowed	57	(21.9)	203	(78.1)	1.4	(0.8, 2.4)
Others (single and divorced)	0	(0)	7	(100.0)	1.7	(0.7, 3.8)
						
Living Arrangements						
Living with others	125	(19.1)	528	(80.9)	1.0	(Reference)
Living alone	12	(14.1)	73	(85.9)	0.7	(0.4, 1.2)
						
Social support						
Not at risk of isolation	98	(16.7)	488	(83.3)	1.0	(Reference)
At risk of isolation	39	(25.7)	113	(74.3)	1.5	(1.1, 2.1)
						
Presence of chronic disease						
None	11	(4.5)	234	(95.5)	1.0	(Reference)
One chronic disease	92	(22.9)	309	(77.1)	5.9	(3.9, 8.7)
More than one chronic disease	34	(36.9)	58	(63.1)	14.2	(7.9, 25.4)
						
Diabetes						
No	69	(13.0)	463	(87.0)	1.0	(Reference)
Yes	68	(33.0)	138	(67.0)	2.5	(1.9, 3.4)
						
Arthritis						
No	82	(13.8)	512	(86.2)	1.0	(Reference)
Yes	55	(38.2)	89	(61.8)	2.8	(2.1, 3.7)
						
Stroke						
No	123	(17.5)	582	(82.5)	1.0	(Reference)
Yes	14	(42.4)	19	(57.6)	2.4	(1.6, 3.7)
						
Depressive Symptomology						
No	81	(14.0)	496	(86.0)	1.0	(Reference)
Yes	56	(34.8)	105	(65.2)	2.5	(1.8, 3.3)
						
Presenting visual acuity						
Normal	89	(15.4)	490	(84.6)	1.0	(Reference)
Mild to moderate visual impairment	39	(31.0)	87	(69.0)	2.4	(1.6, 3.7)
Blind	9	(27.3)	24	(72.7)	3.8	(2.0, 7.1)
						
BMI (weight/height2)						
Normal	51	(19.6)	209	(80.4)	1.0	(Reference)
Underweight	65	(20.4)	254	(79.6)	0.7	(0.5, 1.0)
Overweight	18	(12.5)	126	(87.5)	0.7	(0.3, 1.1)

**Table 5:** Adjusted prevalence ratios for associations between socio-demographic and health related variables and poor physical function among older people in rural Malaysia ^#^

Variables	Physical disability* as dependent variable (n = 738)	Functional Limitation as dependent variable (n = 738)
Age group		
60-64	1.0	1.0
65-69	2.8 (2.2, 3.6)	1.7 (1.3, 2.3)
70-74	4.7 (3.3, 6.8)	2.3 (1.5, 3.4)
≥ 75	7.9 (4.8, 12.9)	3.0 (1.7, 5.2)
		
Female	1.1 (0.7, 2.0)	2.7 (1.2, 6.1)
		
Self reported chronic medical condition Ϯ		
Diabetes Mellitus	1.8 (1.4, 2.3)	1.2 (0.9, 1.5)
Stroke	1.5 (1.1, 2.2)	1.1 (0.7, 1.7)
Arthritis	1.1 (0.8, 1.4)	1.6 (1.2, 2.1)
Presence of Depressive Symptomology Ϯ	1.3 (1.1, 1.8)	2.0 (1.5, 2.7)
		
Presenting visual acuity		
Normal	1.0	1.0
Mild to moderate visual impairment	1.6 (1.1, 2.4)	1.2 (0.7, 2.0)
Blind	2.0 (1.1, 3.6)	1.4 (0.7, 3.0)

* Requiring help in at least 1 out of 10 activities of basic living

# Not significant variables in the full model were omitted from display in the table

Ϯ Reference groups are those without the particular condition

## Discussion

We investigated the functional status of older Malaysians and our findings can be summarised as follows. First, the prevalence of physical disability using the 10 ADL items was 25%, for the 6 item ADL it was 14%, and for the 5 item ADL it was 11%; the prevalence of functional limitation was 20%. Second, physical disability (10 item ADL, 6 item ADL, and 5 item ADL) and functional limitation increased with age, were more common in women than men and among Indians than among Malays and Chinese. Thirdly, variables independently associated with physical disability were advanced age, presence of diabetes, and presence of stroke, depressive symptomology and visual impairment. Advancing age, being female, presence of arthritis and having depressive symptomology were significantly associated with functional limitation

### Comparison with previous studies in Malaysia

There have been several previous studies of physical disability among older people in Malaysia [3,26-32]. Differences in the way disability was measured and sample characteristics make comparisons with our study difficult. However, a population based study of 300 older people in Bangi, Selangor, in 2004 used the 10 item Barthel Index and found that 23% of older people aged 60 years and above were dependent in at least one ADL; similar to the results in our study[28]. A population-based study in 2002 of 223 older people in Sepang, Selangor, using the 10 item Barthel Index found that 16% of older people aged 60 years and above were dependent in at least one ADL. A study done by Siddah et al using the 2003 Mental Health and Quality of Life of Older Malaysians Survey assessed self-reported functional limitations, using two questions " do you have any difficulty in climbing stairs?" and "do you have any difficulty in sitting and standing?". Overall, 13% reported difficulty in climbing stairs, 18% of women and 8% of men. Overall, 10% reported difficulty in sitting and standing, 13% women and 6% of men.

### International comparisons of prevalence of physical disability

We assessed physical disability using the Barthel Index which consists of 10 items of activity in daily living.. Based on the 10-item Barthel Index, a study in Singapore found a much lower disability prevalence than in our study: 11% in people aged 60 years and above (9% when age-standardized to our population sample), 14% in people aged 65 and above and 26% among those aged 75 and above. In our study in rural Malaysia, disability rates in these same groups were twice as high: 25%, 37% and 52% respectively [33]

Comparison with studies in other countries is difficult due to use of different ADL measurements; however, narrowing ADL disability to receiving help for at least one of five ADL items (eating, bathing, dressing, transferring and toileting) or six ADL items (walking, eating, bathing, dressing, transferring and toileting) allows reasonable comparison across some studies.

Using the six item ADL index, the prevalence of physical disability in our study was 20%, which is much higher than the United States' National Long Term Care Survey, 13% (9% when age-standardized to the Malaysian population sample) [7]. However, the prevalence of six item ADL disability among r people age 60 years and above [12], and among people age 75 years and above, in Latin America and the Caribbean appears to be similar to the prevalence rates in our Malaysian study[13].

Using the five item ADL index, for people aged 65 years and above, the prevalence rates in our Malaysian study (16% prevalence of disability) again appear to be much higher than those reported in more developed countries:- 6% in Canada (5% when age-standardized), 10% in France (7% when age-standardized), 14% in Italy (9% when age-standardized) and 11% in Sweden (7% when age-standardized) [7]. Disability prevalence rates in Malaysia appear comparable to rates in other developing countries: For example, among people aged 65 and older, the prevalence of 5 item ADL disability in Sri Lanka was 10% (17% when age standardised) which is almost identical to the 16% prevalence in our study [18]. Among people aged 60 years and over the prevalence of 5 item ADL disability was 11% in our study, similar to 12% in India (10% when age standardized) [15], and 8% in Shanghai, China[19].

There are few possible reasons why Malaysia's disability prevalence is higher than those reported from the developed countries. Firstly, the prevailing socio-demographic differences between Malaysia and the wealthy industrialised countries; the low educational levels in our older cohort (89% have no form of education or low education level), a figure higher than the developed country[34] and higher proportion of older people in Malaysia are still economically active. The 2000 Malaysian census reported that 45% of people in the 60-64 age groups worked in the agriculture related occupation. Secondly, the deeply rooted Asian culture stresses that older people should be taken care of by their family members. An institutional care is accessible to those who can afford (with regards to private nursing homes) and admission to funded shelter homes is usually the last resort to older people who have no heirs and no shelter on their own or those who are destitute. Thus majority of the older people in Malaysia continue to co-reside with their spouse or other family members. As one of the main reasons for institutionalisation in the western countries is disability in ADL [35], this would explain the higher prevalence of physical disability in our study.

Functional limitations can be measured through self report or performance based[5]. Performance based measurements offers more information as they identify important physical parameters involved in performing daily tasks [5]. Comparison of prevalence across studies is difficult due to differences in the conceptual and measurement of functional limitation used. Using the Tinetti Performance Oriented Mobility Assessment Tool, the overall prevalence of functional limitation among Italian aged 75 years and above was 21%, much lower than our prevalence rate of 48% [36]. Older Italian men and women showed similar prevalence of functional limitation 31% and 28% respectively [36]. Our results showed differently, for the same age group, older women reported much higher prevalence of functional limitation (55%), than men (35%).

### Gender and ethnic differences

Women in all age groups showed higher prevalence of physical disability and functional limitation than men. We also found that the gender differences widened with increasing age. Most previous studies have found that women have higher levels of physical disability and functional limitation than men [37-43]. Disadvantages resulting from limited education may contribute to the greater physical disability and functional limitation burden experienced by our older women. Lack of education is often associated with low income and poverty, lower standards of living, unhealthy lifestyle behaviour, unhealthy diet and less frequent use of health and medical care services. Low socioeconomic status has been shown to be associated with physical disability [44] and more women (39%) than men (5%) in our study had no education. Obesity is associated with many chronic diseases such as diabetes, stroke, heart diseases, and arthritis. Obesity was more frequently seen among older women and underweight among older men in our study.

In terms of ethnic variation, we found that Indians had the highest prevalence of self reported physical disability followed by Malays and Chinese. Our findings are similar to previous international studies on ethnic variation and disability [33,45,46]. The observed differences among our ethnic groups might be attributable to different types of occupational history. We found that 76% of the older Indians had worked as manual unskilled workers compared to the 44% of Malays 52% of Chinese. The Indians had mainly worked in rubber plantations. We also found that Malays (77%) were more likely to have one or more chronic diseases than Chinese (40%) and Indians (33%).

### Health Correlates

We found similar variables to be related to physical disability as in other studies: presence of diabetes mellitus [47], stroke [37,40], arthritis [37,48], depressive symptomology [37,49] and visual impairment [50]. Similar to our findings, living alone[40], poor social support [43], were not associated with physical disability.

We found significant associations between functional limitations and advanced age, being female, having arthritis and having depressive symptomology. Living alone, poor social support, being overweight or underweight, presence of diabetes mellitus, stroke and visual impairment were not associated with functional limitation. Our results correspond with previous findings for advanced age [51], female gender [51], presence of arthritis [52] and depressive symptomology [49,52].

This study has some limitations. First, our findings are relevant to community dwelling older adults, similar to most disability studies. This underestimates the prevalence of physical disability and functional limitation as it does not include older people in institutions. Secondly, as with any cross sectional design, it is not appropriate to draw causal inferences between health-related variables and physical disability or functional limitation.

This study has a number of strengths. This is the first study to assess the prevalence and correlates of performance based functional limitation among older Malaysians. We used validated measures of disability that fit with theories of aging. In the Nagi model of disablement, functional limitation precedes disability [53]. Unlike physical disability, functional limitation represents an outcome that is free of the environmental influences. This adds clarity in understanding the dynamics of the pathway from conditions or diseases to disability. Our study population was randomly selected from a geographically defined rural community and we achieved a high participation rate. The age and ethnic group distribution of older people in AGOPHS is consistent with that of older men and women in our target population, according to the 2000 Malaysian Census [25].

## Conclusions

We found that physical disability and functional limitation is common among older people in Malaysia. Our rates appears to be much higher than in developed countries but comparable to other middle-income developing countries. Based on our study findings, the Indians and older people who are underweight should be given priority for additional studies focused on reducing or preventing disability in this population. Our results also show that women, the oldest old, those with chronic diseases, depressive symptomology and visual impairment are at greatest risk of disability and functional limitation. These findings are important for targeting appropriate prevention and intervention strategies. It enables our rural health care professionals to identify older people at risk of developing physical disability and functional limitation. As a result, these people could be referred to interventions aimed at reducing poor physical function such as health education, visits to the homes of high-risk individuals and physical activity programmes for community-living older people, such as taichi.

## Competing interests

The authors declare that they have no competing interests.

## Authors' contributions

NNH: study concept, chief investigator, designing research protocol, data analysis, interpretation of data and writing manuscript. AB, IM contributed in conceptualizing the research and data collection. RGC, VN and AB contributed in critically editing the manuscript. All authors read and approved the final manuscript.

## Acknowledgements

This work was supported by the Fundamental Research Grant Scheme (FRGS), from the Ministry of Higher Education, Malaysia. Dr. Noran N. Hairi's work on this study was supported by the Public Service Department (PSD) of Malaysia. The authors would like to express their appreciation to Dr. Siti Halimah Shaikh and all health care providers of Masjid Tanah Health Clinic, Ministry of Health Malaysia, for their contributions to this research.

## Pre-publication history

The pre-publication history for this paper can be accessed here:

http://www.biomedcentral.com/1471-2458/10/492/prepub
